# Foll Detection of *Toxoplasma gondii* in sheep and goats blood samples by PCR-RFLP in Urmia

**Published:** 2013

**Authors:** Mousa Tavassoli, Mohammad Ghorbanzadehghan, Bijan Esmaeilnejad

**Affiliations:** 1 *Department of Pathobiology, Faculty of Veterinary Medicine, Urmia University, Urmia, Iran; *; 2 * Graduate from Faculty of Veterinary Medicine, Urmia University, Urmia, Iran.*

**Keywords:** Goat, PCR-RFLP, Sheep, *Toxoplasma gondii*, Urmia

## Abstract

Infection by the protozoan parasite *Toxoplasma gondii*, is widespread in humans and many other warm-blooded animals. More than half billion of world human population has serum antibodies to *T.* *gondii* and Sheep and goats are more widely infected with *T. gondii*. *T. gondii* infection can be diagnosed indirectly with serological methods and directly by polymerase chain reaction (PCR), hybridization, isolation and histology. A total number of 124 goats and 113 sheep blood samples were collected from Urmia region and PCR was used for detection of the pathogenic protozoan *T. gondii* using B1 gene. The targeted B1 gene is highly conserved in all *T. gondii* strains and is multiple copy genes whit in the *T. gondii* genome. The method used for the characterization of *T. gondii *strains implied digestion with *Alu*I restriction enzyme of the fragments amplified. The results indicated three positive sheep (1.26%) with one RFLP patterns. The results indicated that the same strain of *T. gondii* has infected sheep in the region.

## Introduction

*Toxoplasma gondii* is an obligatory intracellular parasite that infects a wide variety of warm-blooded animals and human beings.^1^ Cat is the final host of the parasite and is of high importance in its life cycle because only cat sheds the oocytes of the parasite. Human beings could be infected through incidental ingestion of the oocytes, consumption of water or contaminated food with the oocytes and undercooked or raw meat containing cysts. *Toxoplasma* infection in human beings resulted in immunocompromise and abortion or severe clinical signs in fetus or neonate.^1^ Epidemiological findings show that ingestion of oocytes through water and food consumption is the main route of transmission in pregnant women.^2^ Positive serum samples rises with aging and there is no gender susceptibility. Seroprevalence of infection in pregnant women with *T. gondii wa*s reported to be 44.8%, 20.1% and 19.2% in Ilam,^3^ Isfahan^4^ and Sabzevar,^5^ respectively. Also, sero-prevalence of *T. gondii *in sheep and goat is reported to be 24.7% and 15.8% in Kerman,^6^ 24.5% to 32.5% and 19.25% to 17.7% in Iran,^7^^,^^8^ respectively.

Sabin-feldmen dye test is a simple and specific method to detect *T. gondii* but the main disadvantages of the test are the requirement of live parasites and accessory factor from blood donors.^9^ Complement fixation test (CFT) is rarely used for routine laboratory investigations due to its poor sensitivity.^9^ The indirect hemagglutination test (IHA) is simple to perform but the test has failed to detect antibodies at an early phase of infection.^9^ Direct agglutination test is simple and cost effective but it can give false positive results with the serum samples of heart transplant patient with the cytomegalovirus infection.^9^ Immunofluorescence test (IFT) lacks in specificity for the detection of IgM antibodies as it gives false positive or false negative results in some cases.^9^ Enzyme linked immuno-sorbent assay (ELISA) bears high sensitivity, specificity, reproducibility and reliability as compared to other serological methods.^9^ Western blot test is generally used to characterize the *T. gondii* antigen but the diagnostic value based on detection of only antigen is still uncertain because of the diversity of human infection and the variability of pathogenicity of strains.^9^ Therefore, we used PCR technique.

Genetic studies have recently proposed three typical types for* Toxoplasma* genotypes^10^ including Type I, II, and III. The classification is based on their characteristics in electrophoresis of iso-enzymes, PCR-RFLP, random amplified polymorphism DNA (RAPD).^10^^-^^16^ Different strains of *T. gondii *have different pathogenicity in various animals (e.g. type I is more pathogen in mice).^15^^,^^17^ For successful treatment, it is important to determine association among pathogenicity and type of disease and strains of the parasite.^18^ Genetic analysis of strains has demonstrated that proliferation of *T. gondii* is mainly clonal, asexual and single parent, while sexual reproduction exceptionally occurs between different strains in wild types.^15^ Many studies on analysis of isolated types from mice and *in vitro* cultures have demonstrated that type II occurs more common than other genotypes.^5^^,^^13^^,^^19^^-^^22^

To our knowledge, there are no published reports on detection and differentiation of *T. gondii* in small ruminants in Iran. Thus, present study aimed to detect of *T. gondii* in sheep and goat by PCR and differentiation of *T. gondii* strains by RFLP.

## Materials and Methods

In the present study, 124 and 113 blood samples were taken from goats and sheep, respectively. The samples were taken randomly from Urmia abattoir and suburban farms of Urmia between January and April, 2010. DNA was extracted using a DNA purification kit (Fermentas, Berlin, Germany) according to the manufacturer’s instructions. The PCR was done on 50 µL total reaction volume including 5 µL of 10x PCR buffer [70 mM Tris-HCl (pH 8.8), 200 mM (NH_4_)So_4_, 0.1% Tween 20], 2 mM MgCl_2_, 250 µM of each of the four deoxynucleotide triphosphate, 1.25 U Taq DNA polymerase (Fermentas), 50 pmol of each primers (Tox4 3-'CGCTGCAGGGAGGAAGACGAAAGTTG-5**'**) and Tox5 5-'CGCTGCAGACACAGTGCATCTGGATT-3**'**)^19^ and 5 µL of extracted template DNA. Amplification of parasite DNA was done in thermocycler (Model CP2-003, Corbett Research, Sydney, Australia). DNA polymerization was performed as follows (33 cycles): primary denaturation of the samples was performed in 94 ˚C for 7 min denaturation in 94 ˚C for 1 min, annealing in 55 ˚C for1 min, extension in 72 ˚C for1 min and final extension in 72 ˚C for 10 min. DNA positive control for *T. gondii* was provided from Pasteur institute in Iran. Distilled water served as a negative control. Polymerase chain reaction product was run using 1.5% agarose gel PCR product was digested by using* Alu*I enzyme, according to instructions. Show three bands including 364, 87 and 78 bp.

## Results

**Fig 1:**
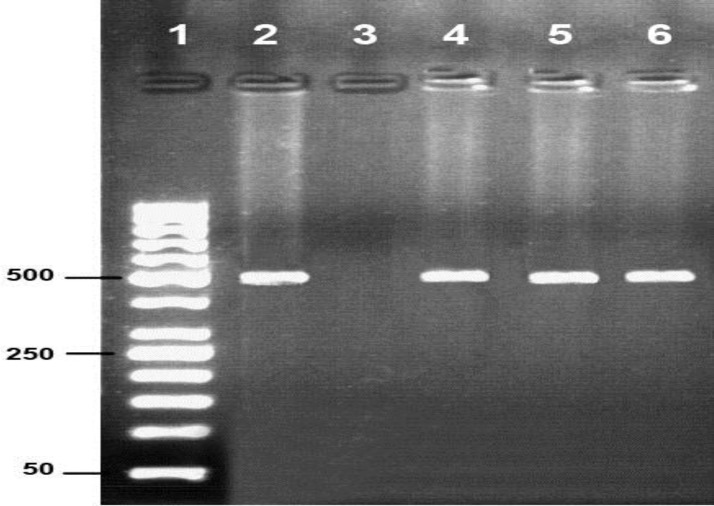
PCR product (529 bp) of B1 gene amplified from infected sheep with* T. gondii*: Lane 1: 50 bp marker, Lane 2: positive control, Lane 3: negative control, Lane 4-6: positive samples

**Fig 2:**
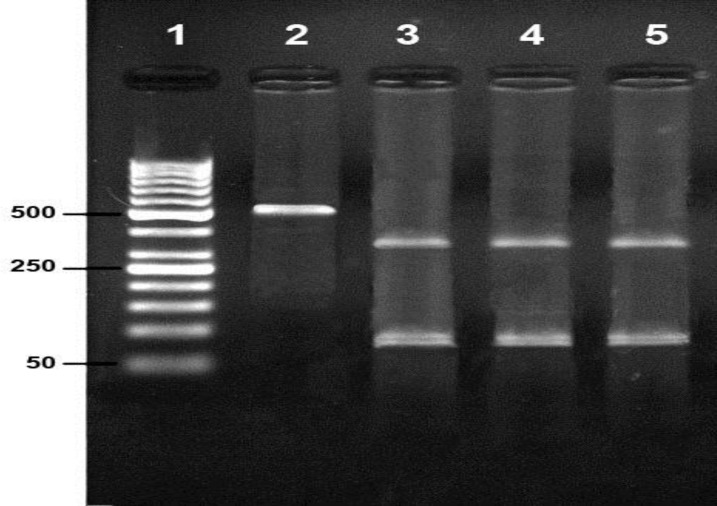
PCR-RFLP pattern of B1 gene amplified from infected sheep with* T. gondii*: Lane 1: 50 bp marker, Lane 2: PCR product, Lane 3-5: RFLP pattern.

Following tracking of PCR products three samples (1.26%) taken from sheep were positive and for goats negative (Fig. 1). RFLP patterns showed that the isolated three samples belonged to the same strain (Fig. 2). 

## Discussion

Serologic methods are standard to detect toxoplasmosis.^23^ In these cases to confirm the diagnosis cell culture, laboratory animal inoculation and/or application of molecular techniques like PCR are employed.^24^ PCR in which a segment of DNA genome of *T. gondii *is detectable, because of adequate sensitivity and specificity, is preferred to other techniques. Getting immediate results is also another advantage.^25^ PCR has been used to detect *T. gondii *in tissue.^26^ There are many reports on comparison of various techniques on diagnosis of *Toxoplasma *infection in different parts of the world.^1^^,^^22^^,^^27^^-^^30^ PCR is very efficient in early diagnosis of toxoplasmosis.^23^ Blood sample is the most available sample required to perform PCR in diagnosis of animal and human cases. However, diagnosis of toxoplasmosis using blood samples gives different results.^23^ These differences are associated with duration of infection and persistence of the parasite in blood. Presence and persistence of *Toxoplama* in blood is related to the strain and route of infection.^23^ Infection of host with tachyzoite, bradyzoite and sporozoite has considerable influence on the time of blood infection and its persistence. For example serum samples of the mice intraperitoneally infected with *Toxoplasma* could be detected after 18 hours by PCR.^31^ Diagnosis of toxoplasmosis in human being is one of applicable technique to detect AIDS patients and infected bone marrow recipients.^32^

In present study, all positive samples belonged to a same strain and infection rate was low. Similar findings have been reported by previous study.^23^ Reports of others were based on detection of antibody in blood samples.^6^^-^^8^ Presence of the hemoparasite in blood occurs in acute phase and because of tachizoites.^21^^,^^33^ In various studies, B1, P30 and DNA ribosomal genes have been used to design and detect *T. gondii*.^11^^,^^25^^,^^34^ B1 gene and DNA ribosomal are appropriate for polymerization in PCR because of presence of their copies in genome of *Toxoplasma*.^35^ B1 gene in genome of *T. gondii *has been repeated 35 times.^11^^,^^36^ Application of B1 gene has got lots of advantages. While primers of B1 gene do not polymerize fungal and bacterial species, primers of P30 gene are less specific than those of B1 gene and it has been demonstrated that primers of P30 gene could polymerize DNA of *Nocardia*^37^ and *Mycobacterium tuberculosis*.^38^ Application of B1 gene has not only high specificity in polymerization of *T. gondii*, but also has high sensitivity in the parasite detection diagnosis.^35^ In contrast with our results, high prevalence (ranging from 14% to 32%.) of *T. gondii* infection in sheep and goats have been previously reported.^6^^-^^8^ Although the results of the present and previous study cannot be compared due to the different of the methods employed. These difference may be associated with cross-reactivity of serological methods with other apicomplexan species.^39^ A similar study, Tavassoli *et al*. reported low infection rate of *T. gondii* and isolated one strain in Urmia, Iran.^23^ Howe *et al*. used blood and CSF samples of the infected patients with toxoplasmosis and 68 samples reported positive with *T. gondii* by PCR method.^14^

In conclusion, PCR-RFLP results were in accordance with the same pattern which related to *Toxoplasma* Type I^24^ and similar strains of *Toxoplasma *which could infect sheep in the region. Because of importance of the parasite, its consequences in human being and economic loss in food animals it is advised to perform PCR in combination with other serologic tests. It is recommended to conduct extended studies regarding infection of human being and other animals in order to detect predominant genotypes of the zoonotic parasite. 
